# Effects of protein–carbohydrate supplementation on immunity and resistance training outcomes: a double-blind, randomized, controlled clinical trial

**DOI:** 10.1007/s00421-016-3520-x

**Published:** 2016-12-27

**Authors:** Fernando Naclerio, Eneko Larumbe-Zabala, Nadia Ashrafi, Marco Seijo, Birthe Nielsen, Judith Allgrove, Conrad P. Earnest

**Affiliations:** 1 Department of Life and Sports Sciences, University of Greenwich, Chatham Maritime, UK; 2 Clinical Research Institute, Texas Tech University Health Sciences Center, Lubbock, TX USA; 3 Faculty of Science Engineering and Computing, Kingston University, London, UK; 4 Exercise Science and Nutrition Laboratory, Texas A&M University, College Station, TX USA

**Keywords:** Immune status, Strength performance, Body composition, Muscle thickness, Blood indices of health

## Abstract

**Purpose:**

To examine the impact of ingesting hydrolyzed beef protein, whey protein, and carbohydrate on resistance training outcomes, body composition, muscle thickness, blood indices of health and salivary human neutrophil peptides (HNP1-3), as reference of humoral immunity followed an 8-week resistance training program in college athletes.

**Methods:**

Twenty-seven recreationally physically active males and females (*n* = 9 per treatment) were randomly assigned to one of the three groups: hydrolyzed beef protein, whey protein, or non-protein isoenergetic carbohydrate. Treatment consisted of ingesting 20 g of supplement, mixed with orange juice, once a day immediately post-workout or before breakfast on non-training days. Measurements were performed pre- and post-intervention on total load (kg) lifted at the first and last workout, body composition (via plethysmography) vastus medialis thickness (mm) (via ultrasonography), and blood indices of health. Salivary HNP1-3 were determined before and after performing the first and last workout.

**Results:**

Salivary concentration and secretion rates of the HNP1-3 decreased in the beef condition only from pre-first-workout (1.90 ± 0.83 μg/mL; 2.95 ± 2.83 μg/min, respectively) to pre-last-workout (0.92 ± 0.63 μg/mL, *p* = 0.025, *d* = 1.03; 0.76 ± 0.74 μg/min, *p* = 0.049, *d* = 0.95), and post-last-workout (0.95 ± 0.60 μg/mL, *p* = 0.032, *d* = 1.00; 0.59 ± 0.52 μg/min, *p* = 0.027, *d* = 1.02). No other significant differences between groups were observed.

**Conclusions:**

Supplementation with a carbohydrate–protein beverage may support resistance training outcomes in a comparable way as the ingestion of only carbohydrate. Furthermore, the ingestion of 20 g of hydrolyzed beef protein resulted in a decreased level and secretion rates of the HNP1-3 from baseline with no negative effect on blood indices of health.

**Electronic supplementary material:**

The online version of this article (doi:10.1007/s00421-016-3520-x) contains supplementary material, which is available to authorized users.

## Introduction

Heavy exercise can lead to significant transient perturbations in immune functions (Walsh et al. 2011b). In athletes, the so-called immunosuppression has been associated with a weakened immune state lasting for up to 72 h after performing a strenuous exercise session (Nieman and Bishop 2006). Consequently, different nutritional strategies, including the combination of carbohydrates with high-quality protein sources, ingested throughout the day (Jones et al. 2015) or during and after workouts or competitions (Naclerio et al. 2015), have been proposed as an effective nutritional countermeasure to exercise-induced immune dysfunction with no detrimental effects on indices of health (e.g., blood chemistry measures) (Antonio et al. 2016). Furthermore, adding protein to augment resistance training outcomes is supported by their capacity to rapidly increase amino acid availability to the working muscles (Wilkinson et al. 2007). Specifically, the post-workout consumption of high-quality proteins would shift the balance in favor of muscle anabolism (Rennie et al. 2004) and, as a consequence, acts to maximize recovery and training effects between workouts (Morton et al. 2015).

Whey and beef protein extracts are high-quality protein sources with a similar amino acid composition to that found in the skeletal muscles (Chernoff 2004; Cruzat et al. 2014). Either whey and beef protein sources include sulfur-containing amino acids, such as cysteine and methionine or taurine that have been associated with an efficient immune status (Reidy and Rasmussen 2016). As a consequence, it could be possible to hypothesize that the ingestion of high-quality protein extracts via the provision of an amino acid would support the immune response in individuals engaged in exhaustive and intense exercise programs.

Defensins, including alpha-defensins, are antimicrobial peptides (AMP) contributing to mucosal host defense providing a broad spectrum of antibacterial and antifungal activity (Wang 2014). The alpha-defensins also known as human neutrophil peptides (mainly HNP1-3) are predominantly found in neutrophils. Within the last 15 years, several investigations have analyzed the response of these salivary markers of humoral immunology in various oral and systemic diseases (Yount and Yeaman 2012). Although some studies have investigated the impact of exercise on humoral immunity (Gleeson 2000), to the best of authors knowledge only two studies have analyzed the HNP1-3 response to exercise (Davison et al. 2009; Gillum et al. 2015) and neither of which have investigated the impact of combining nutritional supplements on the long-term adaptation of humoral immunity. Davison et al. (2009) reported an acute increase in the absolute concentration of the salivary AMP (HNP1-3 and LL-37) in participants having performed a 2.5 h cycling intervention at 60% of *V*O_2max_ while Gillum et al. (2015) observed an acute increase in the level of four AMP (LL-37, HNP1-3, LL-37, L Lactoferrin, and Lysozyme) in participants after 45 min of running at 75% *V*O_2peak_. The post-exercise increase of salivary AMP may to some extent be related to an exercise-induced muscle inflammatory response (Davison et al. 2009). Extraneous dynamic exercise may induce airway inflammation and damage to airway epithelial cell (Davison et al. 2009). Thus, interventions aimed to analyze long-term adaptation on the humoral immunity without side effects on the general health would be of relevant importance for athletes. The aim of the current investigation was to compare the effectiveness of combining an 8-week resistance training program with a commercially available beef hydrolyzed protein powder product, whey isolate or a non-protein, carbohydrate only supplement on resistance training outcomes. The primary outcome for the study was salivary alpha-defensins (HNP1-3) and secondary outcomes included performance, body composition, muscle thickness, and various blood indices of health.

## Methods

### Participants

Forty-two recreationally active college participants (24 male and 18 females) met the requirements to participate in this study. Key criteria for inclusion were: (a) 18–40 year of age, (b) regular recreationally training for at least 2 years with a minimum of 1 month performing resistance training, (c) free from musculoskeletal limitations, (d) agree not to ingest any other nutritional supplements or non-prescription drugs or medication that might affect the immune system or muscle growth as well as the ability to train intensely during the study, and (e) fluent in English. Key criteria used for exclusion were: (a) history of various metabolic conditions and/or diseases; (b) use of a variety of medications, including but not limited to those with androgenic and/or anabolic effects and/or nutritional supplements known to improve strength and/or muscle mass such as creatine, essential amino acid, whey protein, glutamine, dehydroepiandrosterone (DHEA) within 8 weeks prior to the beginning of the study; (d) current use of tobacco products; and/or in the case of female participants ingesting oral contraceptives; and (e) the presence of any orthopedic limitations or injuries. Compliance was confirmed verbally with participants upon arrival to the laboratory.

**Fig. 1:**
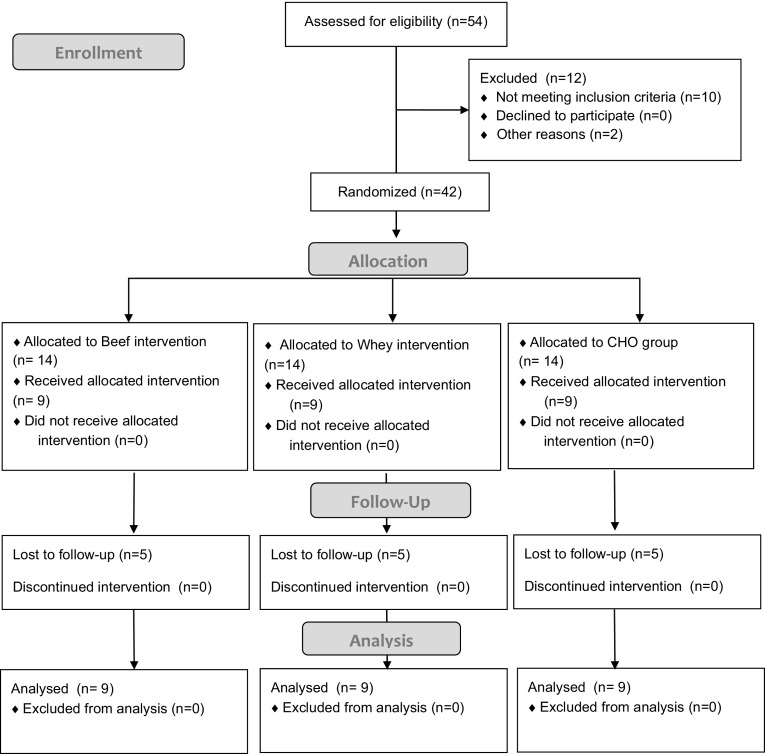
Flow diagram of participants throughout the course of the study

All participants were informed of the potential risks of the intervention before agreeing to comply with the intervention protocol and signed an informed consent. All experimental procedures were conducted in accordance with the Declaration of Helsinki, and approved by the University ethics committee. As summarized in Fig. 1, after assessing for eligibility, 27 of the 42 recruited participants completed all aspects of the study. The study was conducted during the spring and summer of 2015 at the Centre for Science and Medicine in Sport and Exercise University of Greenwich Medway Campus, Kent (UK). Trial Registration: ClinicalTrials.gov, U.S. National Institutes of Health. (Identifier: NCT02425020) on 22nd April 2015.

### Experimental design

This study was a randomized controlled trial with three parallel groups, following a double blind between-participant design. Participants were randomly allocated into three treatment groups: beef protein (*n* = 14); whey protein (*n* = 14) or carbohydrate only (CHO, *n* = 14). Before (test 1) and after (test 2) an 8-week intervention period, measurements of performance, body composition and muscular thickness, total cholesterol, low density lipoprotein (LDL) and high density lipoprotein (HDL) cholesterol, triglycerides, glucose, urea, uric acid, creatine kinase (CK), creatinine, alanine transaminase (AST/GOT), aspartate transaminase (ALT/GPT) and HNP1-3 salivary immune-peptides were assessed. Additionally, saliva was collected before and after the first and the last workout of the 8-week intervention protocol.

After inclusion and before the baseline assessment, participants performed six sessions of familiarization, aimed to minimize any potential learning effects of the training procedures. Following the pre-intervention assessments, participants were matched by gender and resistance training background. Assignment of participants to treatments was performed by block randomization, using a block size of three, and in a double-blind fashion. Initial groups characteristics were as follows: beef: age 25.6 ± 5.7 years, height 1.72 ± 0.09 m, body mass 74.2 ± 17.3 kg; whey: age 25.9 ± 5.9 years, height 1.71 ± 0.10 m, body mass 70.2 ± 11.3 kg; CHO: age 24.9 ± 8.1 years, height 1.70 ± 0.09 m, body mass 70.4 ± 15.3 kg. No significant differences were observed between treatments at inclusion in sample characteristics.

### Dietary supplementation

The three products under study were presented as 20 g sachets of vanilla-flavored powder to be diluted in 250 mL of cold orange juice at each intake. The diluted drinks were similar in appearance, texture and taste and were isoenergetic. The nutritional composition of each product and the amino acids profile of beef and whey proteins are shown in Table 1. Products were taken once a day for 8 weeks (56 total doses per participant). On training days (resistance training or recreational exercise sessions), the supplement was ingested just after training, whereas on non-training days, the product was administered in the morning, before having breakfast.

**Table 1:** Nutritional composition of drinks per intake (20 g of powder plus 250 mL of orange juice)

Nutrient	Beef	Whey	CHO
Energy value (kcal)	184	179	184
Carbohydrates (g)	25	25	45
Lipids (g)	1.54	0.3	0
Proteins (g)	16.4	18	0
Alanine (g)	1.04	1.06	–
Arginine (g)	1.06	0.38	–
Aspartic acid (g)	1.50	2.29	–
Cysteine (g)	0.16	0.48	–
Glutamic acid (g)	2.58	3.34	–
Glycine (g)	1.07	0.34	–
Histidine (g)	0.55	0.31	–
Isoleucine (g)	0.75	1.00	–
Leucine (g)	1.32	1.93	–
Lysine (g)	1.44	1.81	–
Methionine (g)	0.39	0.44	–
Phenylalanine (g)	0.65	0.61	–
Proline (g)	0.81	1.17	–
Serine (g)	0.65	1.05	–
Threonine (g)	0.73	1.44	–
Tryptophan (g)	0.19	0.39	–
Tyrosine (g)	0.52	5.57	–
Valine (g)	0.80	0.98	–
Total EAA (g)	6.82	8.91	–

*EAA* essential amino acids, *CHO* carbohydrates

### Dietary (nutrition) monitoring

A research nutritionist collected dietary habits and explained the proper procedures for recording dietary intake. Each participant completed a 3-day food diary report (2 weekdays, and 1 weekend day). The food diary report was then analyzed using Dietplan 6 software to determine energy and macronutrient content. Participants were instructed to maintain their normal diet throughout the training period. To determine changes and evaluate differences caused by the supplementation protocol, diet composition was analyzed again during the last week of the intervention protocol.

### Measurements

#### Body composition

Body mass (BM) and height were assessed, on a standard scale and stadiometer according the methods described by Ross and Marfel-Jones (1991).

Whole body densitometry was assessed using air displacement via the Bod Pod^®^ (Life Measurements, Concord, CA, USA) in accordance with the manufacturer’s instructions as detailed elsewhere (Dempster and Aitkens 1995). Briefly, the participants were tested wearing only tight fitting clothing (swimsuit or undergarments) and an acrylic swim cap. Volunteers wore exactly the same clothing for all testing. Thoracic gas volume was estimated for all participants using a predictive equation integral to the Bod Pod^®^ software. The calculated value for body density was used in the Siri equation (Siri 1961) to estimate the body composition. A complete body composition measurement was performed twice. If the agreement on percentage of body fat was within 0.05%, the two tests were averaged. If the two tests were not within the 0.05% agreement, a third test was performed and, then, the average of the three completed trials was used for all body composition variables.

#### Muscular thickness

Right-side vastus medialis muscle thicknesses were measured in real time using an Diasus diagnostic ultrasound imaging unit (Dynamic Imaging, Livingston, Scotland UK) coupled to a 50 mm probe at a frequency of 7.5 MHZ while participants were lying supine with arms and legs completely relaxed. The right lower limb was positioned with the knee extended. The probe was placed perpendicular to the skin surface and bone tissues at 80% of the distance between the lateral condyle of the femur and greater trochanter (Bradley and O’Donnell 2002). The probe was coated with a water-soluble transmission gel (Aquasonic 100 Ultrasound Transmission gel) to provide acoustic contact without depressing the dermal surface. Thickness was calculated as the distance between superficial and deep aponeuroses measured at the ends and middle region of each 3.8 cm-wide sonograph.

Three images of each muscle were obtained for each point and the average of the results was calculated. To favor reproducibility, probe placement was carefully noted for reproduction during the other test sessions and the same operator performed all the measurements. To avoid any swelling in the muscles that could disturb the results, images were obtained at least 48 h before and after the program intervention.

#### Training, performance evaluation and control of the intervention compliance

All participants followed the same resistance training routine, three times per week alternated with their habitual recreational recreationally training (games or team sport oriented activity) for a total of 8 weeks (24 resistance training workouts). During the 8 weeks of intervention, participants carried out their workout session late in the afternoon or early evening. After a warm up, the participants performed a total of three circuits involving one set of the following 8 resistance exercises: (1) jump-squat (2) bench press, (3) parallel back squat, (4) upright row, (5) dumbbell alternate lunges, (6) shoulder press, (7) lateral hurdle jumps, and (8) abdominal crunch.

Every exercise included 12 maximum repetitions using the heaviest possible load (except the abdominal crunch that involved 20 repetitions per sets). A minimum rest was permitted in between exercises (only the time required to change from one exercise to the following). Recovery between circuits was 2–3 min. Resistance training sessions were completed in about 30 min. All training sessions were closely monitored to ensure effort, repetitions and intensity established by experienced strength and conditioning coaches. All participants completed all lifts for each exercise. The total load (kg) summarized all repetitions from the first six exercises was considered as the indicator of performance. Although lateral hurdle jumps and abdominals were performed with no external load, performing these exercises at the end of every circuit would impact on general fitness adaptation and the accumulated level of fatigue over the training session. The first and last workouts of the intervention period were used as evaluation sessions. The participants were instructed to refrain from any vigorous activity for 48 h and avoid caffeine ingestion for at least 48 h prior to both assessment sessions. As occurred during the entire training period, the first and last sessions were performed at the same time of the day for the same participant. After completing the first session, each participant was given a batch of products, according to randomization.

Tolerance, collected from adverse events and compliance with product intake (determined by an individual follow up of the participants), was evaluated continuously during the intervention. Only the participants who completed all the 24 training sessions were included in the final analysis.

#### Blood indices of health

Blood chemistry samples were taken before and after the 8-week intervention period. Participants arrived at the physiology laboratory, after a fasting period of 6–8 h, on two separate occasions (1 day before and 1–2 days after completing the 8-week intervention period). Finger-prick capillary whole blood specimens were collected from each subject, under resting conditions, and analyzed using the Cholestech LDX Analyzer for fasting total cholesterol, HDL, LDL cholesterol triglycerides, and glucose, or the Reflotron Plus Clinical Chemistry Analyzer for CK, creatinine, AST/GOT, ALT/GPT, urea and uric acid. Both devices were used according to the manufacturer’s instructions, using test strips (Reflotron Plus Clinical Chemistry Analyzer) or cassette (Cholestech LDX Analyzer).

### Salivary alpha-defensins HNP1-3

#### Saliva collection

Saliva was collected at pre (before and after the first workout) and post (before and after the last workout) 8-week intervention period. At baseline or pre-workout, saliva samples were collected following the blood samples just a few minutes before starting the workout. For the post-workout sample, saliva was collected within 5 min after the exercise cessation.

Participants remained seated, performing minimal movement for 10 min (pre-workout) or 5 min (post-workout) prior to each saliva collection. For all saliva samples, the mouth was rinsed with water at least 5 min before the collection. The participant was requested to swallow to empty the mouth before each sample collection. Unstimulated whole saliva was collected by the spitting method while the participant remained seated, leaning forward and with the heads tilted down (Navazesh 1993). Saliva was collected for 1 min. To avoid circadian variation, saliva samples were collected in between 2 and 7 pm. The collected saliva was weighed to obtain precise flow rate (g/min) (Dawidson et al. 1996). The saliva was stored at −80 °C until further sample treatment and analysis.

#### Saliva analysis

Saliva samples were centrifuged (12,000*g*, 10 min; 4 °C) and the supernatant diluted 1000× with sample dilution buffer. Each sample was analyzed in duplicate with ELISA (Hycult biotech, The Netherlands) following the manufactures instructions. The calibration curve consisted of eight standards, ranging from 0.15 to 10 μg/mL HNP1-3. Absorbance (450 nm) values for the saliva samples were interpolated from calibration standards with a 4-parameter logistic curve (My assays, version 2015). In addition, the alpha-defensins HNP1-3 secretion rates were determined by multiplying their concentration by the flow rate (mL min^−1^).

### Statistical analysis

Sensitivity of the final sample size was calculated assuming a model with three groups and four repeated measures, 0.05 *α* error probability, and 0.80 power (1 − *β*), to ensure adequacy of the study. A descriptive analysis was performed and subsequently the Kolmogorov–Smirnov and Shapiro–Wilk test were applied to assess normality. Sample characteristics at baseline were compared between conditions (beef vs. whey vs. CHO) using one-way analysis of variance (ANOVA). Change from pre to post in performance, body composition, muscle thickness, and blood indices was calculated by subtracting pre- from post-values. Differences in change between conditions were assessed using one-way ANOVA. The changes in the concentration and secretion of HNP1-3 or the saliva flow rates were additionally analyzed using three conditions (beef vs. whey vs. CHO) × four times (pre-workout 1st vs. post-workout 1st vs. pre-workout 24th vs. post-workout 24th) repeated measures ANCOVA, using pre-workout 1st as covariate. Bonferroni-adjusted post hoc analyses were performed when appropriate. Generalized eta squared ($\eta_{\text{G}}^{2}$) and Cohen’s *d* values were reported to provide an estimate of standardized effect size (small *d* = 0.2, $\eta_{\text{G}}^{2}$ = 0.01; moderate *d* = 0.5, $\eta_{\text{G}}^{2}$ = 0.06; and large *d* = 0.8, $\eta_{\text{G}}^{2}$ = 0.14). Significance level was set to *p* < 0.05. Results are reported as mean ± SD unless stated otherwise. Data analyses were performed with Stata 13.1 (StataCorp, College Station, TX, USA).

## Results

Fifteen participants (9 male and 6 female) dropped from the study due to personal reasons, not related with the intervention protocol. Consequently, 27 participants, 15 males and 12 females (5 (55.5%) and 4 (44.4%) per group, respectively) successfully completed the study. The sample was determined to be large enough to detect moderate group–time interactions (*f* = 0.26) through a power analysis. The final composition of the three groups was equivalent at baseline (Table 2).

**Table 2:** Treatment groups description at baseline

Variable	Beef (*n* = 9)	Whey (*n* = 9)	CHO (*n* = 9)	*F*(2,24)	*p*
Age (years)	25.6 ± 5.3	27.6 ± 5.2	24.4 ± 7.1	0.63	0.539
Height (m)	1.72 ± 0.09	1.73 ± 0.1	1.71 ± 0.08	0.14	0.872
Body mass (kg)	69.9 ± 14.35	70.27 ± 12.52	70.83 ± 10.43	0.01	0.988
Fat (%)	23.47 ± 11.53	18.87 ± 7.95	23.42 ± 11.65	0.57	0.574
Fat-free mass (%)	76.53 ± 11.53	81.13 ± 7.95	76.58 ± 11.65	0.57	0.574
Fat (kg)	18.54 ± 12.66	13.43 ± 6.9	16.89 ± 10.12	0.59	0.560
Fat-free mass (kg)	52.47 ± 7.43	56.85 ± 10.58	53.94 ± 10.23	0.49	0.618
Vastus Medialis thickness (mm)	28.24 ± 4.59	31.93 ± 3.09	30.04 ± 4.67	1.75	0.194
Total loaded (kg)	7403.4 ± 1746.5	7942.5 ± 1675.7	7215.6 ± 1637.8	0.45	0.643

All variables are presented as mean (standard deviation); *F* and *p* values were calculated using one-way analysis of variance with 2/24 degrees of freedom

Participants verbally confirmed that they maintained their diet throughout the trial period. Table 3 shows dietary monitoring results, presented as the average daily consumption of carbohydrate, protein, fat (g kg^−1^ day^−1^), and energy (kcal kg^−1^ day^−1^), before and after intervention for the three treatment groups. At baseline, no between-group differences were observed for the amount of protein, carbohydrate, fat or energy intake. However, as a result of the nutritional intervention, all the three groups increased the intake of carbohydrate, while the beef and whey groups raised protein consumption but only beef increased fat intake. All the three treatment groups significantly increased the total energy intake, with no difference between them.

**Table 3:** Analysis of the participant’s diet composition

Treatment	Beef		Whey		CHO	
	Pre	Post	Pre	Post	Pre	Post
Proteins (g kg^−1^ day^−1^)	1.45 ± 0.66	1.70 ± 0.70*	1.47 ± 0.75	1.77 ± 0.92*	1.12 ± 0.56	1.12 ± 0.56
CHO (g kg^−1^ day^−1^)	3.39 ± 1.5	3.76 ± 1.59*	3.16 ± 2.23	3.55* (2.22)	2.59 ± 0.98	3.20 ± 1.04*
Fat (g kg^−1^ day^−1^)	1.20 ± 0.55	1.31 ± 0.56*	1.18 ± 0.40	1.18 ± 0.41	0.91 ± 0.12	0.91 ± 0.12
Energy (kcal kg^−1^ day^−1^)	30.99 ± 12.26	33.77 ± 12.41*	29.93 ± 13.50	31.8 ± 13.85*	23.71 ± 7.21	25.83 ± 7.11*

* *p* < 0.05 significant difference from post- to pre-intervention

### Body composition, muscle thickness, performance and blood indices of health

Combining resistance training with any of the nutrition intervention (beef, whey or CHO) did not produce statistically significant differences between the three treatment conditions in any of the analyzed variables (Table 4). However, beef showed large effect sizes for body mass (*d* = 1.27); total fat-free mass (*d* = 0.75) vastus medialis thickness (*d* = 1.93) and total kg lifted (*d* = 2.16); meanwhile, the CHO group showed large effect sizes for the total kg lifted (*d* = 1.73) and the vastus medialis thickness (*d* = 1.04) and so did the whey treatment only for the total kg lifted (whey *d* = 0.97).

**Table 4:** Differences observed in body composition, muscle thickness, performance and blood markers for the three treatment conditions after the 8-week intervention period

Variable	Beef (*n* = 9)	Whey (*n* = 9)	CHO (*n* = 9)	*F*(2,24)	*p*
Body weight (kg)	1.28 ± 1	0.26 ± 1.29	0.45 ± 1.59	1.53	0.236
Fat (%)	0.17 ± 1.35	−0.41 ± 2.08	−0.1 ± 0.44	0.36	0.705
Fat-free mass (%)	−0.17 ± 1.35	0.41 ± 2.08	0.1 ± 0.44	0.36	0.705
Fat (kg)	0.47 ± 1.07	−0.33 ± 1.62	0.03 ± 0.77	0.99	0.385
Fat-free mass (kg)	0.88 ± 1.18	0.59 ± 0.86	0.42 ± 0.93	0.49	0.618
Vastus medialis thickness (mm)	3.13 ± 1.62	1.57 ± 3.28	1.7 ± 1.63	1.26	0.300
Total loaded (kg)	853.7 ± 395.2	479.1 ± 494.2	763.2 ± 442.3	1.73	0.198
Total cholesterol (mmol/L)	0.09 ± 0.29	0.06 ± 0.46	−0.26 ± 0.41	2.19	0.134
HDL cholesterol (mmol/L)	0 ± 0.35	0 ± 0.18	−0.11 ± 0.23	0.53	0.593
LDL cholesterol (mmol/L)	0 ± 0.52	0.03 ± 0.5	−0.22 ± 0.17	0.95	0.400
Triglycerides (mmol/L)	0.46 ± 0.98	0.17 ± 0.34	0.14 ± 0.72	0.51	0.608
Glucose (mmol/L)	0.48 ± 1.27	−0.14 ± 0.85	0.27 ± 0.93	0.84	0.445
Urea (mg/dL)	3.7 ± 9.7	2.71 ± 3.64	2.44 ± 7.58	0.07	0.931
Uric acid (mg/dL)	−0.33 ± 0.87	−0.18 ± 2.01	0.18 ± 1.06	0.31	0.733
Creatinine (mg)	0.03 ± 0.23	−0.15 ± 0.17	0 ± 0.09	2.61	0.094
AST/GOT (U/L)	−0.13 ± 4.1	1.52 ± 5.53	3.43 ± 7.57	0.82	0.451
ALT/GPT (U/L)	−0.51 ± 2.57	−1.99 ± 8.12	−2.07 ± 9.25	0.13	0.877

All variables are presented as mean (standard deviation); *F* and *p* values were calculated using one-way analysis of variance with 2/24 degrees of freedom

### Salivary alpha-defensins HNP1-3

After adjusting for pre-first-workout using ANCOVA, a significant supplement–time interaction effect for the concentration rates (*F*[6,72] = 2.47, *p* = 0.031, $\eta_{\text{G}}^{2}$ = 0.10), along with statistically significant effect of time (*F*[3,72] = 2.69, *p* = 0.053, $\eta_{\text{G}}^{2}$ = 0.05), but not differences between treatment conditions (*F*[2,23] = 0.65, *p* = 0.533, $\eta_{\text{G}}^{2}$ = 0.014), was observed. No significant interaction (*F*(6,72) = 1.43, *p* = 0.215, $\eta_{\text{G}}^{2}$ = 0.07), treatment condition (*F*(2,23) = 0.54, *p* = 0.591, $\eta_{\text{G}}^{2}$ = 0.008) or time effect (*F*(3,72) = 2.01, *p* = 0.120, $\eta_{\text{G}}^{2}$ = 0.05) was determined on the secretion rates.

**Fig. 2:**
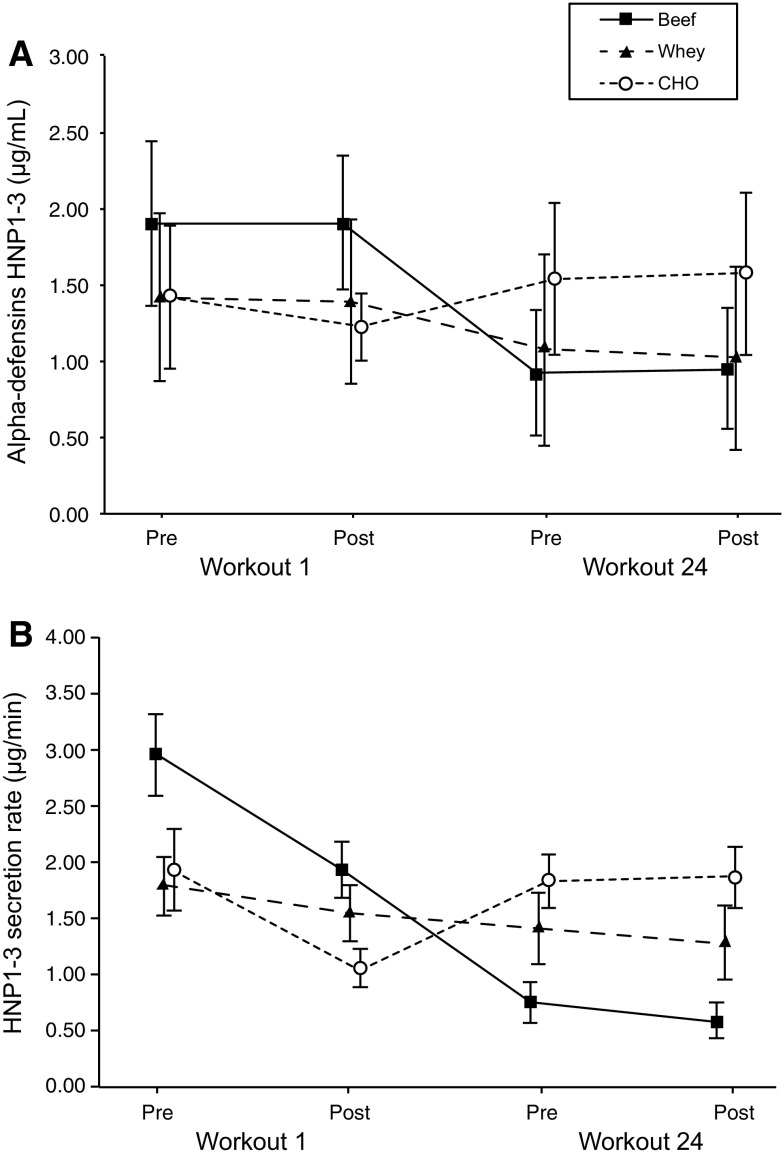
Acute and long-term changes in the concentration (**a**) and secretion rates (**b**) of the alpha-defensins HNP1-3 for the three treatment conditions (mean ± 95% confidence intervals). Statistically significant differences were only found from the baseline levels measured at pre- (before the first workout) to post-intervention, measured at both before and after the last workout for the beef treatment condition for both the concentration (*p* = 0.025 and *p* = 0.049) and secretion rates (*p* = 0.032 and *p* = 0.027), respectively

Bonferroni-adjusted post hoc contrasts to baseline revealed no statistically significant differences between groups at each time point for both the concentration and secretion rates. However, the beef treatment condition showed a statistically significant decrease of the baseline concentration and secretion rates of HNP1-3 from the values measured before performing the first workout (1.90 ± 0.83 μg/mL; 2.95 ± 2.83 μg/min, respectively) of the values determined at post-intervention, before (0.92 ± 0.63 μg/mL, *p* = 0.025, *d* = 1.03; 0.76 ± 0.74 μg/min, *p* = 0.049, *d* = 0.95), and after (0.95 ± 0.60 μg/mL, *p* = 0.032, *d* = 1.00; 0.59 ± 0.52 μg/min, *p* = 0.027, d = 1.02) performing the last workout (Fig. 2a, b).

### Saliva flow rate

No significant interaction (*F*[6,72] = 1.49, *p* = 0.193, $\eta_{\text{G}}^{2}$ = 0.06) or treatment effect (*F*[2,24] = 0.47, *p* = 0.633, $\eta_{\text{G}}^{2}$ = 0.01) was observed. However, a significant time effect (*F*[3,72] = 2.92, *p* = 0.040, $\eta_{\text{G}}^{2}$ = 0.06) was determined. Post hoc analysis revealed a significant intragroup decrease for the beef treatment condition in saliva flow rate baseline levels from the first to the last training session at both pre- (1.336 ± 0.752 vs. 0.589 ± 0.398 mL/min, *p* = 0.036, *d* = 0.99) and post-workout (0.928 ± 0.286 vs. 0.470 ± 0.280 mL/min, *p* = 0.009, *d* = 0.1.14) sessions. No significant differences were found between groups at any time point.

## Discussion

Results suggest that consuming a post-workout carbohydrate–protein (beef or whey) beverage during an 8-week resistance training intervention promotes similar training and body composition outcomes than ingesting only carbohydrate. The most relevant finding of the present investigation was the decrease of the baseline levels and secretion rates of the salivary alpha-defensins HNP1-3 determined after the 8-week intervention protocol in the beef treatment condition. A similar non-significant trend was observed for the whey protein group while no changes were reported for the participants ingesting only carbohydrates. None of the treatment conditions (beef, whey or CHO) affected the normal state of the biochemical blood indices of health or elicited acute changes (pre- to post-workout before and after intervention) of the HNP1-3.

The increase of diet protein consumption in both beef and whey treatment group did not produce any negative effects on the analyzed blood markers of metabolism or hepatic function. The observed response is expected because the amount of daily protein intake for the both protein groups was always within the recommended range of ~1–2 g kg^−1^ (Thomas et al. 2016), Table 3. Indeed, it has been recently indicated that consuming a high protein diet (2.6–3.3 g kg^−1^ day^−1^) over a 4-month period has no negative health-related effect on blood lipids or markers of renal and hepatic function in healthy resistance trained individuals (Antonio et al. 2016).

Data from the present investigation indicate that ingesting a post-workout beverage containing protein with carbohydrate or carbohydrate alone did not influence resistance training responses during the 8-week training period. Overall, the three treatment groups showed similar improvements in body composition, muscle mass and the total kg lifted over the training intervention. However, the beef group seems to elicit a slightly superior body mass gain based on fat-free mass accretion. This appreciation would be supported by the largest effect size observed for the vastus medialis thickness in beef (*d* = 1.93) compared to the other two treatment conditions (whey *d* = 0.48 and CHO *d* = 1.04). Even though previous studies have reported positive effects of canned, minced or beefsteak beef at promoting training outcomes in young (Negro et al. 2014) and elderly individuals (Symons et al. 2009), to the best of the authors’ knowledge, this is the first study to look at the effect of a hydrolyzed beef protein powder extract and compare its effects with those elicited using whey protein and a non-protein isoenergetic nutrient at supporting resistance training outcomes along with analyses of its impact on health and immune markers in young athletes. The total energy provided in the three treatment conditions was almost similar or the amount of protein provided by beef and whey was not sufficient to elicit remarkable differences with respect to the contrast non-protein group. Nevertheless, the present results would still support the premise that daily caloric intake appears to be one of the most relevant factors affecting training adaptations during middle- to long-term exercise interventions (McLellan et al. 2014).

In contrast to previous studies (Davison et al. 2009; Gillum et al. 2015), we did not find acute increases of HNP1-3 after exercise at both before and after the trained intervention. Reasons for this discrepancy could be the nature of the physical exercise. Participants of the present investigation performed eight different resistance training exercises while previous studies have used submaximal cycling (Davison et al. 2009) or running (Gillum et al. 2015) constant intensity protocol. Both aforementioned studies reported acute increases in the concentration of HNP1-3 determined immediately after exercise (Davison et al. 2009; Gillum et al. 2015) or until 1 h compared to pre-exercise values (Gillum et al. 2015). The post-exercise rise in the concentration and secretion rate of AMP could arise from the exercise-induced neutrophilia that occurs in mucosal secretions potentially due to airway inflammation or damage to the airway epithelial cells (Muns 1994). In addition, neutrophilia of the blood can increase HNP1-3 in saliva (Shiomi et al. 1993). These effects have been associated with endurance cyclic exercises such as running or cycling but not with resistance type exercises. Neutrophils contain HNP1-3 in addition to other AMP, such as Lactoferrin and Lysozyme (Gleeson 2007). Endurance continuous exercise has been shown to activate neutrophils possibly causing the release of their contents into the saliva (Pyne 1994), and this is likely the cause of increased salivary levels of AMP after exercise. Even though participants of the current investigation were instructed to perform 12 repetitions of each exercise using the maximum possible amount of load and, consequently, all of them experienced maximum level of fatigue at the end of every session, no acute increase in the salivary concentration of HNP1-3 was determined for both at the beginning and at the end of the 8-week intervention period. Perhaps, the different nature of the applied training protocol alternating exercises involving different muscle groups could have influenced the observed response.

It has been reported that an initial reduction in the concentration of some salivary AMP may be an adaptive response to exercise (West et al. 2010). However, the initial decrease would not be observed in previously well training athletes. Participants of the present investigation were recreationally but regular resistance training individuals and only the participants included in the protein groups (beef and whey, respectively) showed a significant or a trend to decrease the levels of HNP1-3. The aforementioned response would reflect a reduced immune competence with a concomitant increase in the susceptibility to opportunistic infection that has been reported in well-trained athletes (West et al. 2010). However, the clinical relevance of the above-mentioned modification is unknown, since the levels of HNP1-3 measured for the three treatment conditions were similar to those obtained in previous studies (Davison et al. 2009; Kunz et al. 2015). In fact, the salivary levels of AMP including HNP1-3 can vary about 100-fold between individuals. It is, therefore, difficult to define normal-baseline reference ranges (Gorr 2009). Nonetheless, when the individual responses are compared, it is worth noting that all participants but one in the beef group depicted a consistent decrease in the levels of the HNP1-3 from pre- to post-intervention, whereas those in the whey and CHO condition showed different patterns of responses with three participants in the whey and two in the CHO treatment group showing a reduced concentration of HNP1-3 post-intervention.

The HNP1-3 entail a specific group of alpha-defensins that would increase in response to dental caries and the respiratory distress syndrome (Kunz et al. 2015). The after intervention decreased level of HNP1-3 observed in the beef and whey treatment groups could be related to the reduced concentration of carbohydrate ingested during the post-workout period (0.36 ± 0.08 and 0.37 ± 0.06 g kg^−1^ for the beef and whey groups, respectively, vs. 0.65 ± 0.10 g kg^−1^ for the CHO treatment condition). These results would support the notion that only carbohydrate ingestion with no added proteins would probably be the only effective nutritional countermeasure to exercise-induced acute immune dysfunction (Walsh et al. 2011a). Nonetheless, the effectiveness of ingesting carbohydrate at blunting the post-exercises immunosuppression has been mainly observed in acute studies (Walsh et al. 2011a) where the decrease of different immunological markers including some salivary defensins could be interpreted as a transient immune dysfunction with a concomitant higher risk of infection or virus attack (Davison et al. 2009). However, as the drop in the HNP1-3 level observed in the present study was not produced acutely but after an 8-week intervention, it cannot be analyzed as a transient response but as an adaptive medium- to long-term adaptation similar to that observed in other salivary markers in well-trained athletes (West et al. 2010). Considering that HNP1-3 are a marker associated with exercise stress (Kunz et al. 2015), the lower level observed after the intervention in the beef group would be interpreted as a positive adaptive response that allows individuals to reduce the level of stress determined by a given exercise protocol. As previously mentioned, neutrophils are the most abundant white blood cell type and HNP1-3 are the most abundant antimicrobial peptide secreted by neutrophils. Thus, decreased levels of HNP1-3 have also been proposed as an index of reduced risk factor against virus and infection (Mehlotra et al. 2016).

The decreased levels of HNP1-3 resulted after the 8-week intervention program, when participants achieved a better level of performance, which is in line with previous studies that have reported a lower concentration of salivary AMP, including HNP1-3 in better-fit athletes (Kunz et al. 2015). Therefore, the reduced HNP1-3 levels observed after 8 weeks of training could be considered as a normal adaptive response that might have been hasten by the ingestion of the beef protein beverage. Individual differences in training adaptation can be the reason by which some participants allocated in the whey and CHO condition showed a similar post-intervention reduction in the HNP1-3 levels. Additionally, the lower average concentration of HNP1-3 was also related to an increased salivary flow rate observed only for the beef treatment condition. However, differently from the study of Kunz et al., no differences in performance were observed between the three conditions (beef, whey, and CHO) compared to the present study. The higher salivary flow rate, observed in the beef group, may act as a compensatory mechanism to maintain adequate rates of salivary AMP secretion and may, in fact, be indicative of greater parasympathetic nervous system activity (resulting in a large amount of diluted saliva) and vagal tone under the similar training stimulus (Chicharro et al. 1998; Coote 2010) for this particular group.

It is worthy to note that the large variability in the concentration of HNP1-3 observed at rest and in response to exercise observed in the current study is in accordance with others (Davison et al. 2009) who have recognized the variance as a source of limitation for the detection of intervention-induced changes in mucosal parameters, particularly in a parallel group design (Jones et al. 2015). Given the importance of mucosal immune parameters toward host defense (West et al. 2010), further investigation of the effects of post-workout feeding strategies on other salivary AMP (e.g., salivary Lactoferrin and Lysozyme) is warranted.

In conclusion, the present investigation suggests that the ingestion of a carbohydrate–protein (beef or whey) post-workout beverage may support some possible adaptations induced by resistance training in a comparable way as the ingestion of only carbohydrate. Furthermore, the ingestion of 20 g of protein powder, mainly from hydrolyzed beef protein, would also promote an early decrease of the average baseline levels and secretion rates of the salivary alpha-defensins (HNP1-3) with no negative effect on the blood indices of health in recreationally trained college athletes.

## Electronic supplementary material

Below is the link to the electronic supplementary material.

Supplementary material 1 (PPTX 73 kb)

## Abbreviations

AST/GOT: Alanine transaminase
AMP: Antimicrobial peptides
ALT/GPT: Aspartate transaminase
BM: Body mass
CHO: Carbohydrate
CK: Creatine kinase
DHEA: Dehydroepiandrosterone
$\eta_{\text{G}}^{2}$: Generalized eta squared
HDL: High density lipoprotein
HNP1-3: Human neutrophil peptides
LDL: Low density lipoprotein
ANOVA: One-way analysis of variance

## References

[CR1] Antonio J, Ellerbroek A, Silver T, Vargas L, Peacock C (2016). The effects of a high protein diet on indices of health and body composition—a crossover trial in resistance-trained men. J Int Soc Sports Nutr.

[CR2] Bradley M, O’Donnell P (2002) Atlas of musculoskeletal ultrasound anatomy Greenwich Medical Media London

[CR3] Chernoff R (2004). Protein and older adults. J Am Coll Nutr.

[CR4] Chicharro JL, Lucia A, Perez M, Vaquero AF, Urena R (1998). Saliva composition and exercise. Sports Med.

[CR5] Coote JH (2010). Recovery of heart rate following intense dynamic exercise. Exp Physiol.

[CR6] Cruzat VF, Krause M, Newsholme P (2014). Amino acid supplementation and impact on immune function in the context of exercise. J Int Soc Sports Nutr.

[CR7] Davison G, Allgrove J, Gleeson M (2009). Salivary antimicrobial peptides (LL-37 and alpha-defensins HNP1-3), antimicrobial and IgA responses to prolonged exercise. Eur J Appl Physiol.

[CR8] Dawidson IJ, Ar’Rajab A, Melone LD, Poole T, Griffin D, Risser R (1996). Early use of the Gore-Tex Stretch Graft. Blood Purif.

[CR9] Dempster P, Aitkens S (1995). A new air displacement method for the determination of human body composition. Med Sci Sports Exerc.

[CR10] Gillum TL, Kuennen MR, Castillo MN, Williams NL, Jordan-Patterson AT (2015). Exercise, but not acute sleep loss, increases salivary antimicrobial protein secretion. J Strength Cond Res.

[CR11] Gleeson M (2000). Mucosal immune responses and risk of respiratory illness in elite athletes. Exerc Immunol Rev.

[CR12] Gleeson M (2007). Immune function in sport and exercise. J Appl Physiol.

[CR13] Gorr SU (2009). Antimicrobial peptides of the oral cavity. Periodontol.

[CR14] Jones AW, Thatcher R, March DS, Davison G (2015). Influence of 4 weeks of bovine colostrum supplementation on neutrophil and mucosal immune responses to prolonged cycling. Scand J Med Sci Sports.

[CR15] Kunz H (2015). Fitness level impacts salivary antimicrobial protein responses to a single bout of cycling exercise. Eur J Appl Physiol.

[CR16] McLellan TM, Pasiakos SM, Lieberman HR (2014). Effects of protein in combination with carbohydrate supplements on acute or repeat endurance exercise performance: a systematic review. Sports Med.

[CR17] Mehlotra RK, Zimmerman PA, Weinberg A (2016). Defensin gene variation and HIV/AIDS: a comprehensive perspective needed. J Leukoc Biol.

[CR18] Morton RW, McGlory C, Phillips SM (2015). Nutritional interventions to augment resistance training-induced skeletal muscle hypertrophy Front Physiol.

[CR19] Muns G (1994). Effect of long-distance running on polymorphonuclear neutrophil phagocytic function of the upper airways. Int J Sports Med.

[CR20] Naclerio F, Larumbe-Zabala E, Cooper R, Allgrove J, Earnest CP (2015). A multi-ingredient containing carbohydrate, proteins l-glutamine and l-carnitine attenuates fatigue perception with no effect on performance, muscle damage or immunity in soccer players. PLoS One.

[CR21] Navazesh M (1993). Methods for collecting saliva. Ann N Y Acad Sci.

[CR22] Negro M, Vandoni M, Ottobrini S, Codrons E, Correale L, Buonocore D, Marzatico F (2014). Protein supplementation with low fat meat after resistance training: effects on body composition and strength. Nutrients.

[CR23] Nieman DC, Bishop NC (2006). Nutritional strategies to counter stress to the immune system in athletes, with special reference to football. J Sports Sci.

[CR24] Pyne DB (1994). Regulation of neutrophil function during exercise. Sports Med.

[CR25] Reidy PT, Rasmussen BB (2016). Role of ingested amino acids and protein in the promotion of resistance exercise-induced muscle protein anabolism. J Nutr.

[CR26] Rennie MJ, Wackerhage H, Spangenburg EE, Booth FW (2004). Control of the size of the human muscle mass. Annu Rev Physiol.

[CR27] Ross WD, Marflel-Jones MJ, MacDougal JC, Wenger HA, Green HJ (1991). Kineanthropometry. Physiological testing of high performance athlete.

[CR28] Shiomi K, Nakazato M, Ihi T, Kangawa K, Matsuo H, Matsukura S (1993). Establishment of radioimmunoassay for human neutrophil peptides and their increases in plasma and neutrophil in infection. Biochem Biophys Res Commun.

[CR29] Siri WE, Brozek J, Henschel A (1961). Body composition from fluid spaces and density: analysis of methods. Techniques for measuring body composition.

[CR30] Symons TB, Sheffield-Moore M, Wolfe RR, Paddon-Jones D (2009). A moderate serving of high-quality protein maximally stimulates skeletal muscle protein synthesis in young and elderly subjects. J Am Diet Assoc.

[CR31] Thomas DT, Erdman KA, Burke LM (2016). Position of the Academy of Nutrition and Dietetics, Dietitians of Canada, and the American College of Sports Medicine: Nutrition and Athletic Performance. J Acad Nutr Diet.

[CR32] Walsh NP (2011). Position statement. Part two: Maintaining immune health. Exerc Immunol Rev.

[CR33] Walsh NP (2011). Position statement. Part one: Immune function and exercise. Exerc Immunol Rev.

[CR34] Wang G (2014). Human antimicrobial peptides and proteins. Pharmaceuticals (Basel).

[CR35] West NP, Pyne DB, Kyd JM, Renshaw GM, Fricker PA, Cripps AW (2010). The effect of exercise on innate mucosal immunity. Br J Sports Med.

[CR36] Wilkinson SB, Tarnopolsky MA, Macdonald MJ, Macdonald JR, Armstrong D, Phillips SM (2007). Consumption of fluid skim milk promotes greater muscle protein accretion after resistance exercise than does consumption of an isonitrogenous and isoenergetic soy-protein beverage. Am J Clin Nutr.

[CR37] Yount NY, Yeaman MR (2012). Emerging themes and therapeutic prospects for anti-infective peptides. Annu Rev Pharmacol Toxicol.

