# Supplementary material for: Effects of protein–carbohydrate supplementation on immunity and resistance training outcomes: a double-blind, randomized, controlled clinical trial
Source: Eur J Appl Physiol. 2016 Dec 27;117(2):267–77. doi: 10.1007/s00421-016-3520-x (PMC5313575; doi:10.1007/s00421-016-3520-x)
Supplement: Supplementary file 1 — Supplementary material 1 (PPTX 73 kb) [file 421_2016_3520_MOESM1_ESM.pptx]

## Slide 1
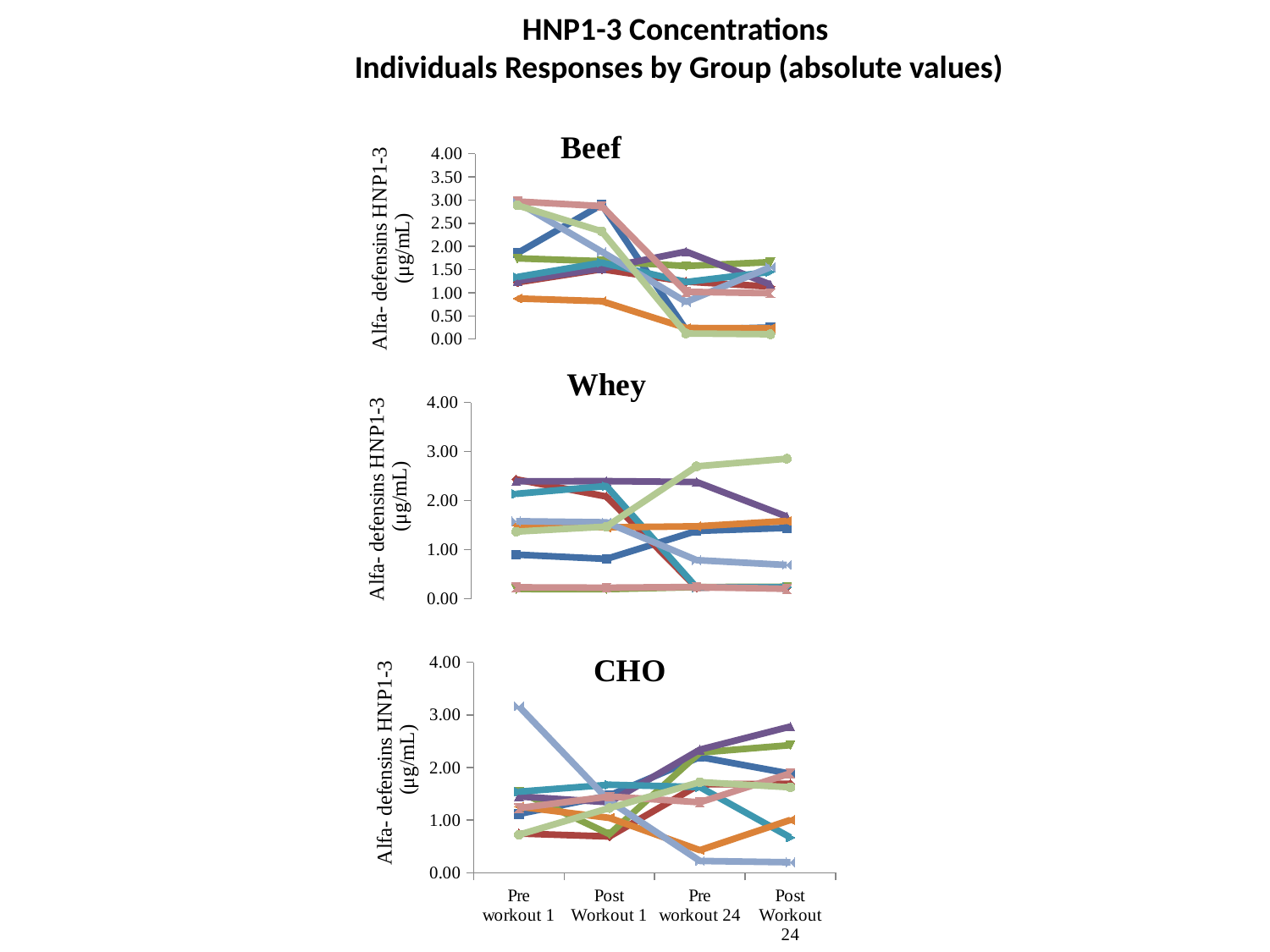

HNP1-3 Concentrations
 Individuals Responses by Group (absolute values)
### Chart: Beef
| Category | | | | | | | | | |
|---|---|---|---|---|---|---|---|---|---|
| Pre workout 1 | 1.856 | 1.224 | 1.742 | 1.253 | 1.34 | 0.8756 | 2.951 | 2.97 | 2.887 |
| Post Workout 1 | 2.9 | 1.506 | 1.682 | 1.523 | 1.65 | 0.81678 | 1.887 | 2.87 | 2.325 |
| Pre workout 24 | 0.201 | 1.2385 | 1.577 | 1.888396 | 1.23 | 0.2384 | 0.8004 | 1.02 | 0.12 |
| Post Workout 24 | 0.25 | 1.133 | 1.661 | 1.177 | 1.45 | 0.2306 | 1.55 | 0.99 | 0.103649 |
### Chart: Whey
| Category | | | | | | | | | |
|---|---|---|---|---|---|---|---|---|---|
| Pre workout 1 | 0.9012 | 2.434 | 0.2003 | 2.394 | 2.139 | 1.503 | 1.58 | 0.2345 | 1.369 |
| Post Workout 1 | 0.8134 | 2.084 | 0.198 | 2.397 | 2.297134 | 1.456 | 1.558 | 0.2272 | 1.47 |
| Pre workout 24 | 1.383 | 0.2281 | 0.2398 | 2.381 | 0.2339 | 1.477 | 0.789 | 0.2418 | 2.698 |
| Post Workout 24 | 1.444 | 0.238 | 0.2471 | 1.676 | 0.238 | 1.585 | 0.689369 | 0.204 | 2.854 |
### Chart: CHO
| Category | | | | | | | | | |
|---|---|---|---|---|---|---|---|---|---|
| Pre workout 1 | 1.118 | 0.7461 | 1.545 | 1.45 | 1.541 | 1.2569 | 3.163 | 1.23 | 0.7227 |
| Post Workout 1 | 1.47 | 0.689369 | 0.7346 | 1.34 | 1.673 | 1.043 | 1.363 | 1.45 | 1.231 |
| Pre workout 24 | 2.2 | 1.68 | 2.285 | 2.34 | 1.628 | 0.4299 | 0.2256 | 1.34 | 1.723 |
| Post Workout 24 | 1.884 | 1.693 | 2.426 | 2.78 | 0.672 | 1.004 | 0.2001 | 1.89 | 1.625 |

## Slide 2
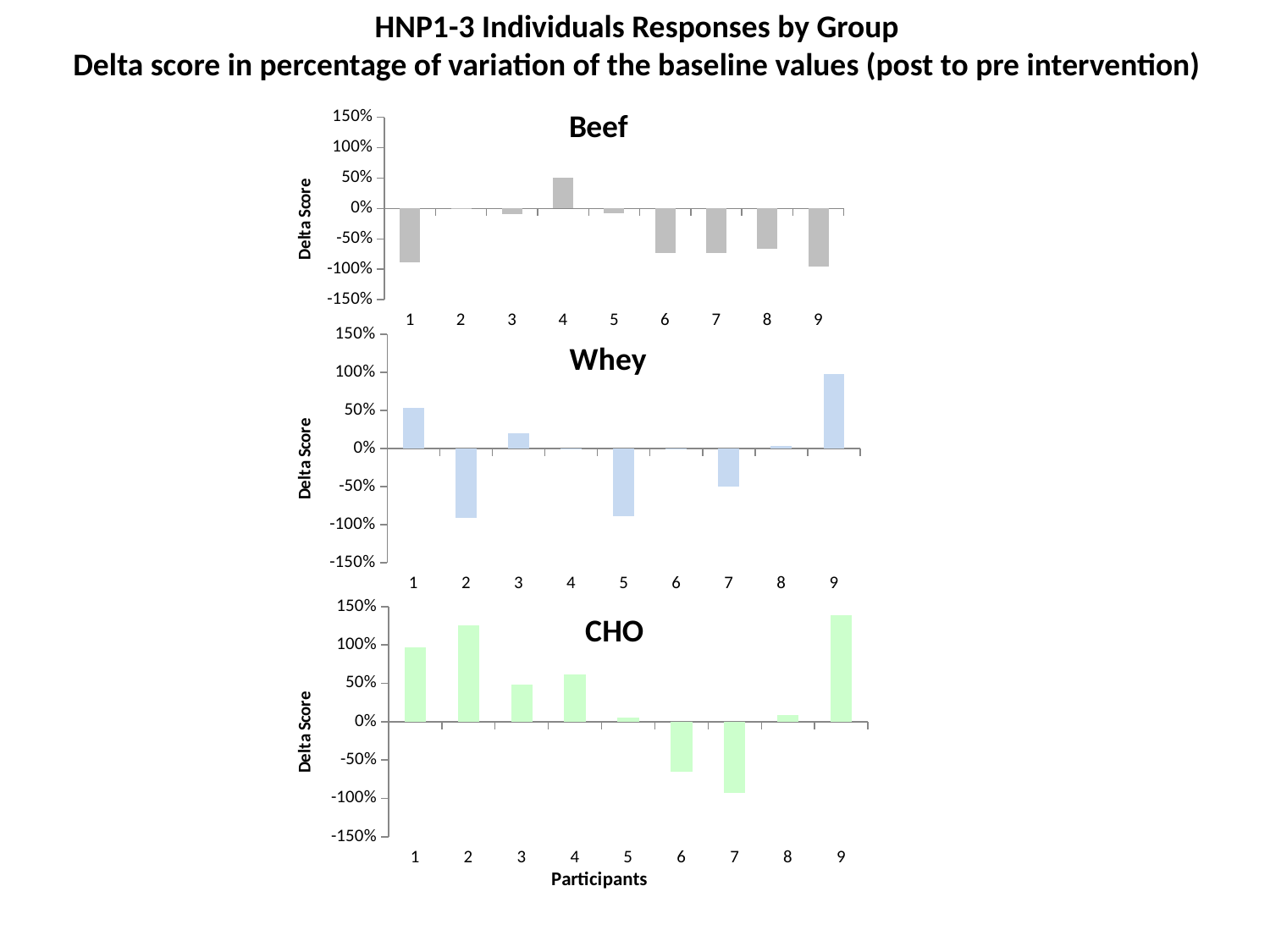

HNP1-3 Individuals Responses by Group
Delta score in percentage of variation of the baseline values (post to pre intervention)
### Chart: Beef
| Category | |
|---|---|
### Chart: Whey
| Category | |
|---|---|
### Chart: CHO
| Category | |
|---|---|
